# Design and Control of a Ferromagnetic Coded Micro-Carrier Biochip Sensor for Multiplex Detection of Antibodies

**DOI:** 10.3390/s110807851

**Published:** 2011-08-11

**Authors:** Rong-Seng Chang, Jang-Zern Tsai, Tung-Yen Li

**Affiliations:** 1 Department of Optics and Photonics, National Central University, 300 Chung-Da Rd., Chung-Li 32001, Taiwan; 2 Department of Electrical Engineering, National Central University, 300 Chung-Da Rd., Chung-Li 32001, Taiwan; E-Mails: jztsai@ee.ncu.edu.tw (J.-Z.T.); ligalike@gmail.com (T.-Y.L.)

**Keywords:** ferromagnetic coded micro-carrier, magnetic force, hybridization

## Abstract

This paper describes a method for producing a novel type of ferromagnetic coded micro-carrier. The ferromagnetic coded micro-carriers are about 200 μm in length, 200 μm in width and 50 μm in thickness, and contain eight code elements with two distinguishable codes (hollow and solid), allowing for 2^8^ unique codes. The code shapes include rectangle, circle, *etc*. Differently shaped coded micro-carriers could carry different antigens for detection of its complementary antibody. These many shapes of coded micro-carriers would be used simultaneously allowing us to make multiple detections for different antibodies at the same time. A molding process is applied for fabrication of the ferromagnetically coded micro-carriers where Fe material (Fe powder mixed with binder) is shaped in many tiny molds to produce the coded shapes used for identification of the bio-molecules. Magnetic force is used to control the movement and location of the ferromagnetic coded micro-carriers to prevent the loss during the hybridization process. The results of image process and analysis system testing are satisfactory. The results of our micro-carrier detection system for two sets of R and B color analysis are proportional to those obtained from ELISA antibody detection.

## 1. Introduction

Recently, the star of biotechnology has been rising. Early theorization of developmental biochip systems [1,2] can be traced back to micro-array biochip and coded micro-carrier systems (for example also see our 2004 article [3] about coded micro-carrier biochips). An increasing number of recent publications and empirical studies have reassessed the positive contributions that code micro-carrier systems can make to biotechnology.

On the whole, though, there has been little published in the literature on how to control the movement of coded micro-carriers. Early attempts [3] at exploring this issue have proved disappointing. In previous methods, the coded micro-carrier container was made by glass tube in which the bottom part was cut out and recovered by a hollowed mesh net to preserve the micro-carriers during the hybridized process. The coded micro-carriers were transferred onto a mesh or into a pipe during the experimental procedure, but some of the coded micro-carriers were lost during the transmission and washing processes. These defects make the whole experimental process inefficient. To overcome these shortcomings, a novel control method for coded micro-carriers is presented. The purpose of this study is to address some of the most important questions contain the collection and the move set of the coded micro-carriers that have arisen in previous research and set forth some explicit parameters: to use magnetic field control and ferromagnetic coded micro carriers for looking for a new control method for coded micro-carriers.

This study focuses on a new coded micro-carrier design and a control method for them [4]. A simple general method is developed. The coded micro-carriers are manufactured from a soft iron (Fe) material, so that magnetic force or magnetic circuit methods can be used to control their movement [5–7]. The practicality of the proposed methodology is demonstrated experimentally. The results could have considerable impact on the development of biochip systems. While research on the control of coded micro-carriers is still in the beginning stages, the findings will also have broad implications in other research areas such as anti-counterfeiting trademarks (a patent is pending).

## 2. Experimental Section

### 2.1. Ferromagnetic Coded Micro-Carrier Manufacturing Process

At the present time, there are two main kinds of biochip systems being used worldwide, the micro-array biochip system, and the coded micro-carrier biochip system. In the past, coded micro-carriers were made utilizing a LIGA-like process [8]. The most common material used for their manufacture was polyethyleneterephalate (PET). The dimensions of coded micro-carriers are on the order hundreds of micrometers, so they are too tiny to be seen with the naked eye. This makes them hard to collect and their movement during the experimental process difficult to control. To solve this problem, we develop a new type of micro-carrier of a ferromagnetic material which could be easily collected and controlled by the application of a magnetic field.

Our new coded micro-carriers or “ferromagnetic coded micro-carriers” are made by a Metal Injection Molding (MIM) process [9–11]. MIM is a process in which small metal parts may be manufactured with the precision shape forming ability of plastic injection molding, and with the performance and material properties. The method for manufacturing the new micro-carriers is described below.

**Figure 1.**
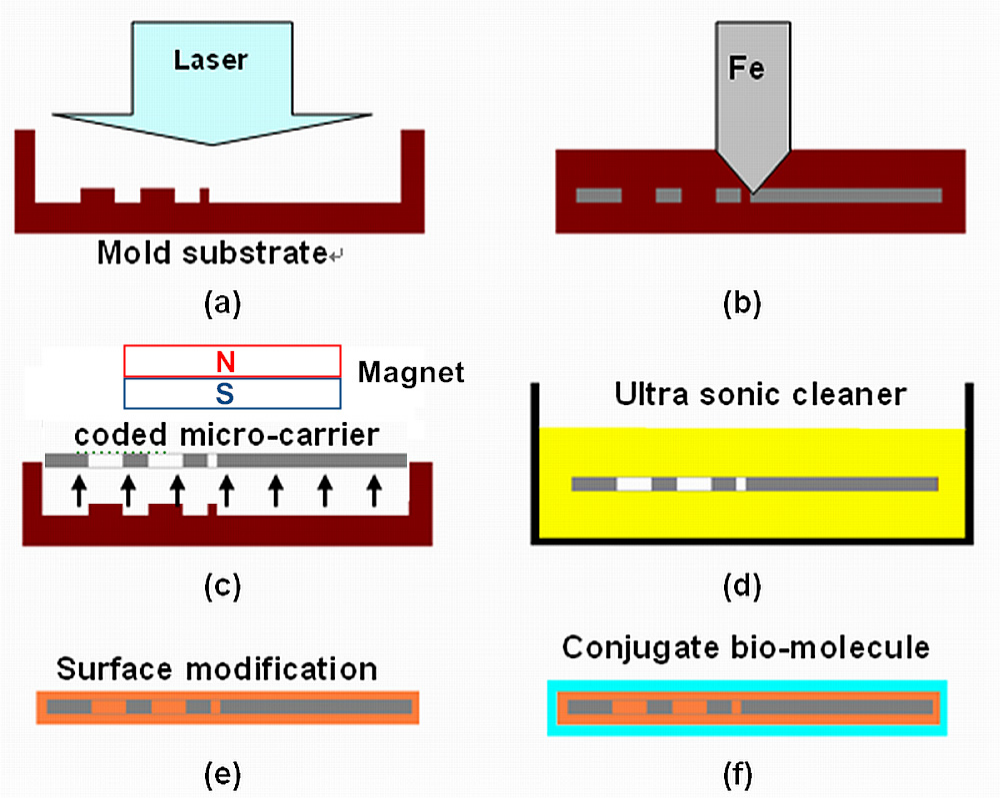
Manufacturing process of the ferromagnetic coded micro-carriers: **(a)** molding; **(b)** injection; **(c)** stripping; **(d)** ultra-sonic cleaning; **(e)** surface modification; **(f)** addition of conjugate bio-molecules.

The code is represented by different shapes such as rectangles, circles, and so on. The fabrication process for creating the coded micro-carriers includes the following steps:

- Molding [Figure 1(a)]: a mold substrate is first prepared on which the contours of the micro-carrier and the coding pattern such as the variable geometry are laser etched.
- Injection [Figure 1(b)]: the Fe material (Fe powder mixed with binder) is injected into the micro-carrier mold by Metal Injection Molding process. In this study, the dimensions of the micro-carriers are as follows: 200 μm in length, 200 μm in width and 50 μm in thickness.
- Stripping the micro-carriers [Figure 1(c)]: when the ferromagnetic coded micro-carrier is done, it is removed by the magnetic ejection method from the magnet. We use magnet to attract the ferromagnetic coded micro-carriers.
- Ultra-sonic cleaning [Figure 1(d)]: to avoid the influence of pitting and residues caused by the manufacturing process on the data analysis results, a sonic cleaner is used to clean the surfaces of the micro-carriers for about 10 min.
- Surface modification [Figure 1(e)]: it is necessary to change the surface properties of the ferromagnetic coded micro-carrier so that proteins or DNA can be more strongly bound by bio-molecular reactions.
- Addition of conjugate bio-molecules [Figure 1(f)]: a layer of bio-molecular binding material is then coated onto the ferromagnetic coded micro-carriers. The particles combined with the corresponding bio-molecules to produce ferromagnetic coded micro-carriers.

The ferromagnetic coded micro-carriers can be controlled by using an applied magnetic field. They hybridize with unknown bio-molecules, combining to form conjugate bio-molecules and micro-carriers. The labeling color and the coding of the micro-carriers can be detected microscopically.

### 2.2. Magnetization of the Ferromagnetic Coded Micro-Carriers

**Figure 2.**
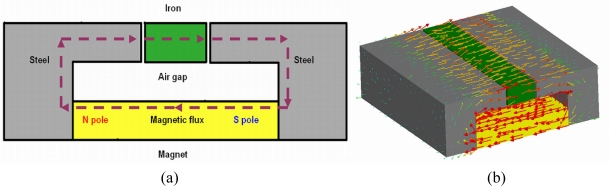
Schematic representation of the magnetization process: **(a)** 2D plot; **(b)** 3D simulation.

There are some crystalline materials that, because of their crystallization structure and microscopic organization, exhibit the property of ferromagnetism, for example, Fe, Ni, and so on. A ferromagnetic material such as iron can be temporarily magnetized by the addition of a magnetic field. Figure 2 illustrates the magnetization process of a ferromagnetic material. After removal of the additional magnetic field, the magnetization of the ferromagnetic material will decrease rapidly, or even be lost. By taking advantage of this feature, we are able to design a magnetic circuit device to place and move the ferromagnetic coded micro-carriers.

### 2.3. Surface Modification Method and Biospecific Interaction

Using only the method described above we could not bind proteins successfully to the surface of the ferromagnetic coded micro-carrier, without changing its surface properties [12–14]. To simulate a glass surface, a SiO_2_ layer is coated onto the surface of the micro-carrier by an immersion method. During the period of immersion, a magnetic circuit is applied to generate a magnetic field, to force the ferromagnetic coded micro-carriers to stay clustered in the same position, and avoid their disappearance during the transfer operation from one glass tube or well to the other.

After the application of the SiO_2_ film layer by the immersion process, the ferromagnetic coded micro-carriers are ultrasonically dried with extra dry N_2_.

**Figure 3.**
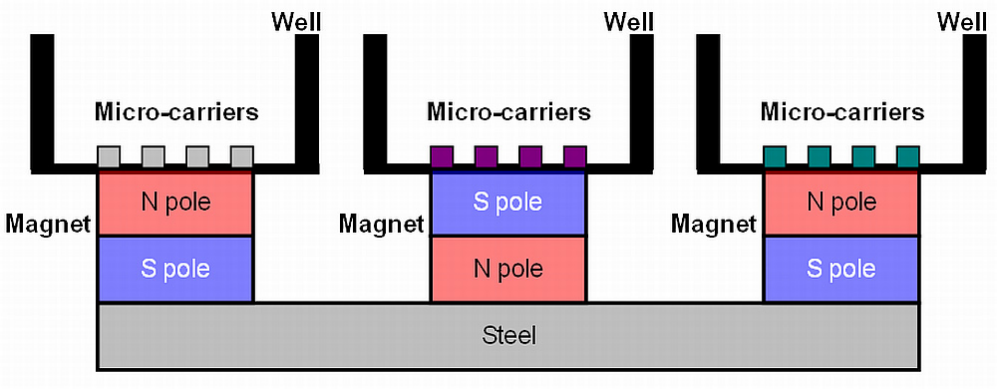
Schematic illustration of the magnetic circuit device.

The hybridization process and interaction of the ferromagnetic coded micro-carriers with the proteins, step (f) in Section 2.1, is described in more detail as follows:

Step 1: A few ferromagnetic coded micro-carriers (10–15 pieces) are placed into each well of a 96-well plate, and 100 μL GAL1 [15–17] added, as well as a phosphate-buffer saline (PBS, as a negative control) to each well and then shaken overnight at 4 °C.
Step 2: The liquid is removed and each well washed three times with a 200 μL PBST (PBS containing 0.1% Tween 20) for 10 min.
Step 3: The ferromagnetic coded micro-carriers are moved to clean wells by the application of a magnetic force.
Step 4: The PBST is removed and 100 μL of a blocking buffer (5% Bovine Serum Albumin in PBST) added to each well before shaking for 1 h at room temperature.
Step 5: The blocking buffer is removed and 100 μL of the first antibody (anti-galectin-1 monoclonal antibodies) with several different dilutions (0, 20, 80, 140, 200, 300 μg/well) added to each well before shaking for 1 h at room temperature.
Step 6: The first antibody is removed as well as any unbounded first antibody by washing three times with PBST for 10 min.
Step 7: One hundred μL of the secondary antibody (alkaline phosphatase-conjugated anti-mouse Ig) [diluted 2,000-fold in a blocking buffer] are added to each well, before shaking for 1 h at room temperature.
Step 8: The secondary antibody, as well as any unbounded secondary antibody is removed by washing 3 times with PBST for 10 min.
Step 9: The BCIP/NBT alkaline phosphatase substrate solution in each well is removed. Reactions are facilitated under light-avoiding conditions until a dark purple color appears on the ferromagnetic coded micro-carriers (about 30 min).
Step 10: The BCIP/NBT substrate is removed, and ddH_2_O is added to stop the reaction.
Step 11: Microscopic images of the ferromagnetic coded micro-carriers can be obtained with a microscope and a CCD camera. The features and characteristics of the images are then analyzed by a computer.
Step 12: During steps 1 to 10, a magnetism field is generated by an NdFeB magnet to keep the ferromagnetic coded micro-carriers in the wells, decreasing the loss of micro-carriers during the experiments (See Figure 3).

The procedures in steps 1 through 11 are repeated without the ferromagnetic coded micro-carriers, and the experimental data recorded for each well (TEST FILTER: 570 nm) with an ELISA reader (DYNATECH MRS, Great Britain).

### 2.4. Multiplexed Detection of Unknown Antibodies by Use of Ferromagnetic Coded Micro-Carriers

The hybridization process and multiplexed detection of ferromagnetic coded micro-carriers with unknown antibodies, are described in more detail below:

Step 1: First choose two kinds of ferromagnetic coded micro-carriers with different codes (coding A and coding B). Place a few ferromagnetic coded micro-carriers into separate wells of a 96-well plate. 100 μL of GAL1 are added to a well containing coding A ferromagnetic coded micro-carriers. 100 μL of HSP27 are added to another well containing coding B ferromagnetic coded micro-carriers before shaking overnight at 4 °C.
Step 2: The liquid is removed and each well washed three times with a 200 μL PBST (PBS containing 0.1% Tween 20) for 10 min.
Step 3: The two kinds of ferromagnetic coded micro-carriers are moved to a clean well by the application of magnetic force.
Step 4: The PBST is removed, and add 100 μL of a blocking buffer (5% Bovine Serum Albumin in PBST) added to each well before shaking for 1 h at room temperature.
Step 5: The blocking buffer is removed and 100 μL of the unknown antibody added into the well before shaking for 1 h at room temperature.
Step 6: The unknown antibody, and any unbounded unknown anti-body is removed by washing three times with PBST for 10 min.
Step 7: One hundred μL of the secondary antibody (alkaline phosphatase-conjugated anti-mouse Ig) [diluted 2,000 fold in a blocking buffer] are added to the well, before shaking for 1 h at room temperature.
Step 8: The secondary antibody and any unbounded secondary antibody is removed by washing three times with PBST for 10 min.
Step 9: The BCIP/NBT alkaline phosphatase substrate solution is added into the well, to facilitate reactions under light-avoiding conditions until a dark color appears on the ferromagnetic coded micro-carriers (about 30 min).
Step 10: The BCIP/NBT substrate is removed, and ddH_2_O added to stop the reaction.
Step 11: Microscopic images of the ferromagnetic coded micro-carriers can be obtained with a microscope and a CCD camera. The features and characteristics of the images are then analyzed on the computer.
Step 12: During steps 1 to 10, an NdFeB magnet is utilized to keep the ferromagnetic coded micro-carriers in the well, and decrease micro-carrier loss during the experiments.

### 2.5. Testing Platform and Data Analysis

**Figure 4.**
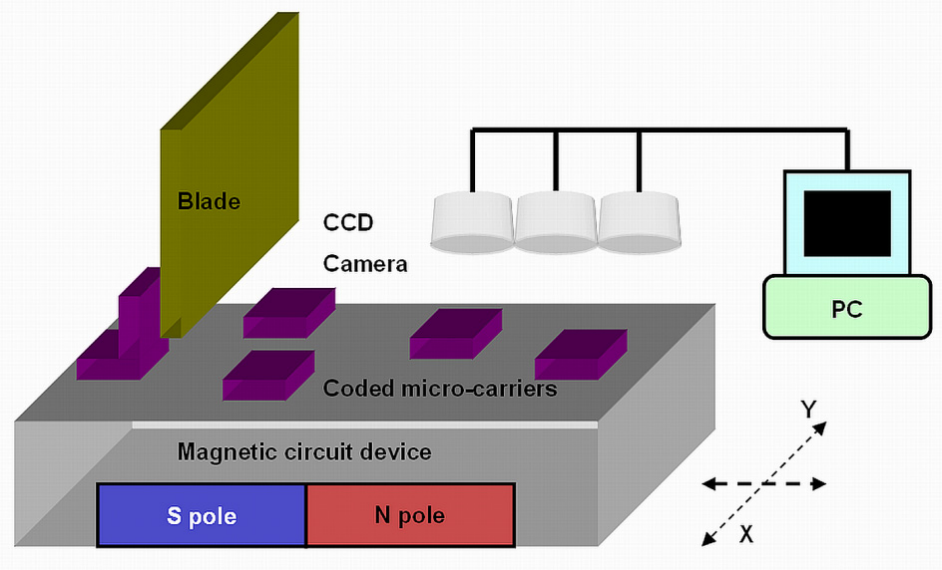
Schematic illustration of the testing platform.

We developed a platform for holding the ferromagnetic coded micro-carriers after hybridization, as a method for preserving and testing the bio-molecular information. A schematic illustration of the experimental apparatus used in the present study is shown in Figure 4. The apparatus is comprised of four parts, a microscope with CCD camera, an x/y direction table, non-magnetic blades and a magnetic circuit device. Ferromagnetic coded micro-carriers can be removed from the glass tubes or wells mentioned above using a magnetic tweezers, than placed on the x/y direction table.

The magnetic circuit device creates a magnetic field on the x/y table. The magnetic force keeps the ferromagnetic coded micro-carriers on the surface of the x/y table. To avoid the problem of the micro-carriers overlapping, non-magnetic blades are used to let a single layer of ferromagnetic coded micro-carriers pass through, while sweeping off the overlapping micro-carriers.

In this testing system, there is more than one microscope focused on the x/y table, so that images of the coded micro-carriers can be caught simultaneously by multiple-microscopes and CCD cameras. The use of multiple-microscopes increases the testing area on the x/y table as well as the testing speed. This multiple microscope testing system can collect large amounts of coded micro-carrier information. The improved contrast image fusion is used for information analysis [18]. The image fusion method is applied to fuse two images of the same hybridized coded micro-carriers from two different filters into one better image.

After the image fusion process, we obtain clearer images of the coded micro-carriers. The composition of the color distribution on the coded micro-carriers is then analyzed. The analysis is carried out using the MATLAB software package. First, the contours and positions of the coded micro-carriers are computed. Next, color distribution information is calculated. Both qualitative and quantitative data analyses are performed.

Enzyme-Linked ImmunoSorbent Assay (ELISA) assays are used to examine the relationships between variable conditions. Two different techniques were used in this investigation. Finally, the experimental data obtained from ferromagnetic coded micro-carriers after image processing and the ELISA assays are compared and evaluated.

### 2.6. The Coding Rule of Ferromagnetic Coded Micro-Carriers

**Figure 5.**
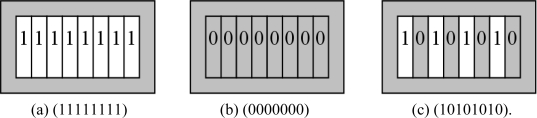
Barcode coding **(a)** (11111111); **(b)** (0000000); **(c)** (10101010).

**Figure 6.**
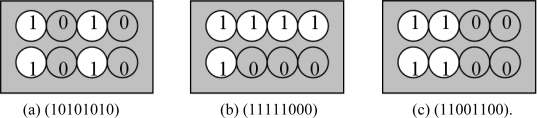
Circular-hole coding **(a)** (10101010); **(b)** (11111000); **(c)** (11001100).

Here we have different coding methods indicated in Figures 5 and 6. We use the barcode coding and circular-hole coding:

Barcode coding which has been shown in Figure 5(a–c), hereby we have shown eight rectangular holes (1 × 8 matrix), each hole location symbolized by “0” (for solid, gray area) and “1” (for hollow, white area). In barcode coding method, there are 2^8^ unique codes.

Circular-hole coding which has been shown in Figure 6(a–c), hereby we have shown eight circular holes (2 × 4 matrix), each hole location symbolized by “0” (for solid, gray area) and “1” (for hollow, white area). In circular-hole coding method, there are 2^8^ unique codes.

**Figure 7.**
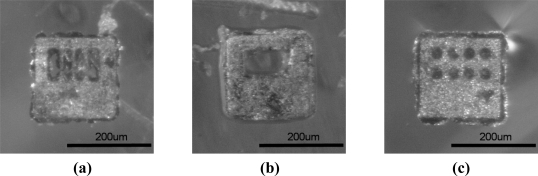
Ferromagnetic coded micro-carriers with different codes: **(a)** rectangular (10101010); **(b)** rectangular (11111000); **(c)** circular (11111111).

Figure 7 shows the shapes of the differently coded ferromagnetic coded micro-carriers which include rectangles and circles. Differently shaped coded micro-carriers carry different antigens with which to detect their complementary antibody allowing for the simultaneous detection.

## 3. Results and Discussion

**Figure 8.**
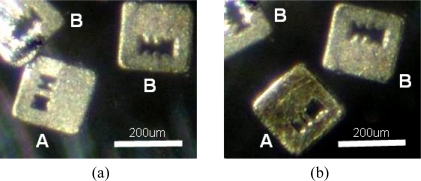
Multiplex detection of ferromagnetic coded micro-carriers with unknown antibody: **(a)** before hybridization; **(b)** after hybridization.

**Figure 9.**
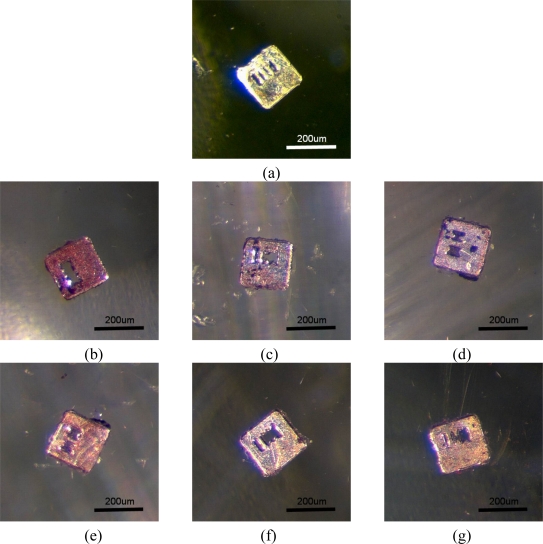
Photographs of ferromagnetic coded micro-carriers with different dilution ratios of 1°Ab after hybridization: **(a)** negative control; **(b)** 0; **(c)** 20; **(d)** 80; **(e)** 140; **(f)** 200; **(g)** 300.

In Figure 8(a), two kinds of ferromagnetic coded micro-carriers with different codes (coding A and coding B) in the same well are shown. At this time, the color of these two kinds of ferromagnetic coded micro-carrier is the same. After the hybridization process with unknown antibodies [see Figure 8(b)], we see a change in the color of the A coded micro-carrier. This shows us that the unknown antibodies are anti-galectin-1 monoclonal antibodies, and that we can make multiple detections of antibodies at the same time. Multiple-microscopes with CCD cameras are used to obtain images of the micro-carriers after completion of the hybridization processes with different experimental variables (see Figure 9). The MATLAB software is used for analysis and arrangement of the information obtained from the images, which includes red and blue color components. Table 1 highlights the relations between the color presentation and the density ratio of the 1°Ab.

**Figure 10.**
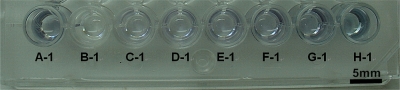
Photograph of results of ELISA assays.

The wells and results of the ELISA assays are illustrated in Figure 10. It should be noted that the experimental parameters in sections A-1 to H-1 are different. A 570 nm test filter is used, and data measurements made by the ELISA reader are shown in sections A-1 to H-1. The experimental conditions and data are presented in Table 2.

Overall, the results are very positive, being as expected in terms of the relationship between the digital image processing and ELISA measurement results. Both types of experimental results show a clear and strong relationship between the 1°Ab dilution ratio and the measurement data. The ELISA measurement data and the dilution ratio of 1°Ab are shown to be positively correlated with one another. Looked at in another way, the gray levels of the color component on the coded micro-carriers are significantly moderately negatively related to the 1°Ab dilution ratios. The gray level increases in value with decreasing dilution ratio. This can be explained by an increase in the density of 1°Ab, so the color on the coded micro-carriers shows a darkening phenomenon.

Therefore, we transfer the gray level expression by 255 minus the gray level value (*G*). The density of antibody (*I*) can be expressed as follows:

(1) I=K•(255−G)

where *I* is the density of the antibody, *K* is the proportional constant and *G* is the gray level value.

**Figure 11.**
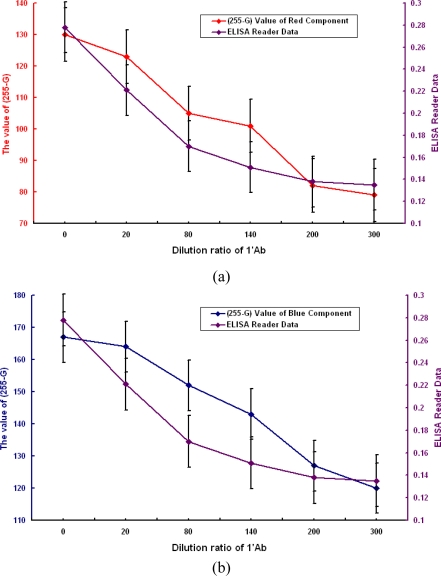
Results and comparison of the (255-G) value of images and ELISA reader data: **(a)** red component; **(b)** blue component.

From the above and Figure 11, we find that the two sets of numbers match. The test results obtained by the image processing and analysis systems show that our micro-carrier detection method detects two sets of R and B color analysis which are proportional to ELISA antibody detection.

## 4. Conclusions

This study builds a method for producing a novel type of coded micro-carrier. Different shaped coded micro-carriers can carry different antigens which are used to detect their complementary antibodies. In short, many shape coded micro-carriers can be used for multiple detections of different antibodies at the same time. We carried out experiments to build the ferromagnetic coded micro-carriers and a test platform. Magnetic fields are used to control the movement and location of the ferromagnetic coded micro-carriers during the hybridization process without loss. The image processing and analysis system results are satisfactory: our micro-carrier detection analysis consisting of two sets of R and B colour are matched with ELISA antibody detection which is proportional to our R and B color analysis results.

**Figure 12.**
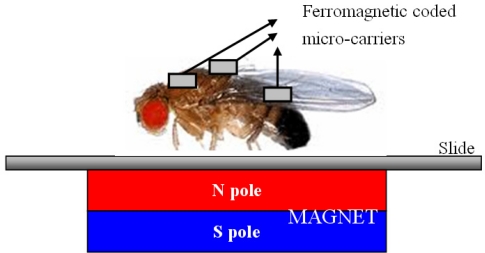
The diagram of ferromagnetic coded micro-carriers *in vivo* by testing the skin of *Drosophila*.

There are some multiple functional chips in existence such as, micro-array systems. However, our chips can be used for *in vivo* on-site experiments: stationary platforms for bio-detection (as shown in this paper) or for mobile magnetic field-directed application (in the future), which a conventional micro-arrays can not do. Our ferromagnetic coded micro-carriers can be fixed in place by the use of magnetic force which also decreases the chip loss during the hybridization process, washing process and so on. In the future, we will inject the ferromagnetic coded micro-carriers (or nano-carriers) into vascular and guide them by outside magnetic field to the location of interesting area [5–7]. For example, we can apply ferromagnetic coded micro-carriers *in vivo* by testing the skin of *Drosophila* (see Figure 12). We also can detect nano-carriers by Transmission X-ray Microscope (TXM) [19].

Our ferromagnetic coded micro-carriers were analyzed by image processing with CCD cameras and computer. A simple and cost effective solution for bio-molecular applications is developed. The ferromagnetic coded micro-carrier requires no expensive equipment, offers great flexibility, and more capability to test unknown biomaterials.

## Figures and Tables

**Table 1.** Measurement results of ferromagnetic coded micro-carriers by image processing.

**Figure**	**GAL1**	**1°Ab Dilution ratio**	**2°Ab Dilution ratio**	**Gray level of red Component**	**Gray level of blue Component**
9(a)	PBS (−)	20	2,000	198	147
9(g)	100 μg/mL	300	2,000	176	135
9(f)	100 μg/mL	200	2,000	173	128
9(e)	100 μg/mL	140	2,000	154	112
9(d)	100 μg/mL	80	2,000	150	103
9(c)	100 μg/mL	20	2,000	132	91
9(b)	100 μg/mL	0	2,000	125	88

Figure 9(a) shows the negative control.

**Table 2.** Measurement results from ELISA reader.

**Section**	**GAL1**	**1°Ab Dilution ratio**	**2°Ab Dilution ratio**	**ELISA reader Data**
A-1	100 μg/mL (+)	20	2,000	0.193
B-1	PBS (−)	20	2,000	0.061
C-1	100 μg/mL	300	2,000	0.135
D-1	100 μg/mL	200	2,000	0.138
E-1	100 μg/mL	140	2,000	0.151
F-1	100 μg/mL	80	2,000	0.170
G-1	100 μg/mL	20	2,000	0.221
H-1	100 μg/mL	0	2,000	0.278

A-1 is the positive control, B-1is the negative control.
